# Exposure to environmental contaminants and folic acid supplementation intergenerationally impact fetal skeleton development through the paternal lineage in a rat model

**DOI:** 10.3389/ftox.2022.881622

**Published:** 2022-09-27

**Authors:** Phanie L. Charest, Emmanuel Tessougue, Maryse Lessard, Pauline M. Herst, Pauline Navarro, Sarah Kimmins, Jacquetta M. Trasler, Amanda J. MacFarlane, Marie-Odile Benoit-Biancamano, Janice L. Bailey, Mathieu Dalvai

**Affiliations:** ^1^ Department of Animal Sciences, Faculty of Agricultural and Food Sciences, Université Laval, Quebec City, QC, Canada; ^2^ Centre de Recherche en Reproduction Développement et Santé Intergénérationnelle (CRDSI), Université Laval, Quebec City, QC, Canada; ^3^ Department of Nutrition, Faculty of Agricultural and Food Sciences, Institute of Nutrition and Functional Foods, Université Laval, Quebec City, QC, Canada; ^4^ Department of Pharmacology and Therapeutics, Faculty of Medicine, McGill University, Montreal, QC, Canada; ^5^ Department of Animal Science, Faculty of Agricultural and Environmental Sciences, McGill University, Montreal, QC, Canada; ^6^ Departments of Pediatrics, Human Genetics and Pharmacology and Therapeutics, Research Institute-McGill University Health Center, McGill University, Montreal, QC, Canada; ^7^ Nutrition Research Division, Health Canada, Ottawa, ON, Canada; ^8^ Groupe de Recherche En Pharmacologie Animale du Québec (GREPAQ), Department of Pathology and Microbiology, Faculty of Veterinary Medicine, Université de Montréal, Sainte Hyacinthe, QC, Canada

**Keywords:** development, fetus, folic acid, persistent organic pollutants, prenatal exposure, skeleton, POPs, DOHaD (development origins of health and disease)

## Abstract

Persistent organic pollutants (POPs) are ubiquitous in the environment, which is of concern since they are broadly toxic for wildlife and human health. It is generally accepted that maternal prenatal folic acid supplementation (FA) may beneficially impact offspring development, but it has been recently shown that the father's exposures also influence the health of his offspring. Bone is an endocrine organ essential for whole-body homeostasis and is susceptible to toxicants. Herein, we tested the hypotheses that prenatal paternal exposure to POPs induces developmental bone disorders in fetuses across multiple generations and that FA supplementation attenuates these disorders. We used a four-generation rat model, in which F0 founder females were divided into four treatment groups. F0 females were gavaged with corn oil or an environmentally-relevant POPs mixture and fed either a control diet (2 mg FA/kg), or FA supplemented diet (6 mg FA/kg) before mating and until parturition (four treatments in total). After the birth of the F1 litters, all F0 females and subsequent generations received the FA control diet. Staining with alcian blue and alizarin red S of male and female fetal skeletons was performed at Gestational Day 19.5. Paternal direct and ancestral exposure to POPs delayed bone ossification and decreased the length of long limb bones in fetuses. Maternal FA supplementation did not counteract the POPs-associated delayed fetal ossification and reduced long bone length. In conclusion, prenatal paternal POPs exposure causes developmental bone abnormalities over multiple generations, which were not corrected by maternal FA supplementation.

## Introduction

Notorious for their resistance to degradation and semi-volatility, persistent organic pollutants (POPs), such as polychlorinated biphenyls (PCBs), and dichlorodiphenyltrichloroethane (DDT), remain present in the environment despite restrictions by the 2001 Stockholm Convention (The World Health Organisation, 2011; Wohrnschimmel et al., 2016). Although the presence of several POPs has since declined, new POPs are emerging and climate change enhances the release of sequestered POPs, as the warming environment facilitate the liberation of iced trapped POPs (Hung et al., 2016; Houde et al., 2019). Their lipophilic nature allows POPs to bioaccumulate and biomagnify in the food chain, which is of concern since they are broadly toxic for wildlife and human health, and several are considered endocrine disruptors, such as DDT and PCBs (Zoeller et al., 2012). Studies have reported neurobehavioural, immunological, reproductive, cardiovascular, endocrine, and carcinogenic effects following antenatal and/or life exposure to POPs (Jayaraj et al., 2016; Weihe et al., 2016).

Although offspring health is traditionally associated with the maternal environment (Sharp et al., 2018), paternal exposures also influence the development of subsequent generations (Veenendaal et al., 2013; Braun et al., 2017; Xavier et al., 2019). Indeed, our team has demonstrated that ancestral exposure of male rats to an environmentally relevant POPs mixture impairs reproductive and metabolic function for multiple generations (Herst et al., 2019; Lessard et al., 2019; Navarro et al., 2019).

In contrast, knowledge about the consequences of POPs exposure on skeletal development is scarce. Bone tissue produces hormones such as fibroblast growth factor 23 (FGF23) and osteocalcin (Cappariello et al., 2016), and is thereby implicated in the regulation of physiological processes such as glucose and energy homeostasis (Fulzele et al., 2010), phosphate homeostasis (Lang et al., 2018), brain development (Oury et al., 2013), immune response (Weitzmann, 2017), and male fertility (Oury et al., 2011). Dysregulation of these bone-mediated functions could, therefore, be detrimental to human health. Since bone is vulnerable to toxicants and endocrine-active substances (Agas et al., 2018), the impact of POPs exposure on skeletal development is of interest. Herein, we thus test the hypothesis that prenatal paternal exposure to environmentally-relevant POPs disrupts skeletal development and that the phenotype is maintained across multiple unexposed generations in a rat model.

Further, a therapeutic approach to counteract environmental POPs is desirable (Hennig et al., 2012). Folic acid (FA) is essential for normal foetal brain, skull and spine development (Morse, 2012). To reduce neural tube defects (NTDs), FA supplementation during the periconceptional period is recommended for women at risk of becoming pregnant, and many countries have implemented mandatory FA fortification of grain products to increase FA intake (De Wals et al., 2007; Berry et al., 2010). As a methyl donor for one-carbon metabolism it plays an important role in cell proliferation, DNA synthesis and in establishment of the epigenome through its influence on DNA and histone methylation (Crider et al., 2012). Altered maternal and paternal one-carbon metabolism has been associated with birth defects, cardiometabolic disorders and cancer (Crider et al., 2012; Guéant et al., 2013). Our laboratory has also shown that prenatal exposure to POPs results in altered sperm parameters, induces negative pregnancy outcomes, and alters the DNA methylation profile as well as sperm miRNA. (Maurice et al., 2018; Herst et al., 2019; Lessard et al., 2019; Maurice et al., 2021). Therefore, we tested a second hypothesis that maternal FA supplementation counteracts detrimental effects of prenatal paternal exposure to POPs and thus protects subsequent unexposed generations.

## Materials and methods

### Persistent organic pollutant mixture

The POPs mixture, previously described (Lessard et al., 2019), was designed to resemble the contaminant composition and concentration present in northern Canadian populations (Muir, 1995; Anas et al., 2005). The experimental dose, therefore, represents an environmentally relevant exposure and is reported as 500 µg PCBs/kg body weight plus other POPs in relative proportions. The detailed composition of the mixture is presented in Table 1.

**TABLE 1:** Composition of POPs mixture (Anas et al., 2005; Lessard et al., 2019).

Compound	Cas no.	Origin^a^	% In mixture	Dose (µg/kg body weight/3x week)
Aroclor and congener neat mix^b^		AccuStandard	32.4	500
Technical chlordane	57-74-9	AccuStandard	21.4	330.3
Dichlorodiphenyldichloroethylene (*p,p’*-DDE)	72-55-9	Sigma-Aldrich	19.3	297.8
Dichlorodiphenyltrichloroethane (*p,p’*-DDT)	50-29-3	SigmaAldrich	6.8	104.9
Technical toxaphene	8001-35-2	AccuStandard	6.5	100.0
*α-*hexachlorocyclohexane (α-HCH)	319-84-6	Sigma-Aldrich	6.2	95.7
Aldrin	309-00-2	Sigma-Aldrich	2.5	38.6
Dieldrin	60-57-1	Sigma-Aldrich	2.1	32.4
1, 2, 4, 5-tetrachlorobenzene	95-94-3	Sigma-Aldrich	0.9	13.9
Dichlorodiphenyldichloroethane (*p, p'*-DDD)	72-54-8	Sigma-Aldrich	0.5	7.7
*β-*hexachlorocyclohexane (*β-*HCH)	319-85-7	Sigma-Aldrich	0.4	6.2
Hexachlorobenzene (HCB)	118-74-1	AccuStandard	0.4	6.2
Mirex	2385-85-5	Sigma-Aldrich	0.2	3.1
Lindane (γ-HCH)	58-89-9	Sigma-Aldrich	0.2	3.1
Pentachlorobenzene (PeCB)	608-93-5	Sigma-Aldrich	0.2	3.1

a AccuStandard Inc., (New Haven, Connecticut); Sigma-Aldrich Inc., (St Louis, Missouri).

b Mix containing PCBs: Aroclor 1260 (58.9%); Aroclor 1254 (39.3%); 2,4,4′-trichlorobiphenyl (PCB 28; 1%); 2,2′,4,4′-tetrachlorobiphenyl (PCB 47; 0.8%); 3,3′,4,4′,5-pentachlorobiphenyl (PCB 126; 0.02%), and 3,3′,4,4′-tetrachlorobiphenyl (PCB 77; 0.004%).

### Folic acid diets

As described previously (Herst et al., 2019; Lessard et al., 2019; Navarro et al., 2019), the animals were fed diets based on the AIN-93G formula, designed to promote growth, pregnancy, and lactational phases (Reeves et al., 1993) and the diets differed only by their level of FA. The FA control diet contained 2 mg FA/kg diet, reflecting the AIN-93G diet, and the supplemented FA diet contained 6 mg FA/kg diet (# 110700 and #117819 respectively by Dyets, Inc., Bethlehem, PA). The FA control diet was assumed to approximate the recommended dietary intake for human adults (0.4 mg/day), whereas the supplemented FA diet approximated intake by a Canadian woman in the postfortification era from FA-enriched foods and prenatal supplements (≈1.2 mg/day) (Swayne et al., 2012a).

### Animal model and breeding

**FIGURE 1:**
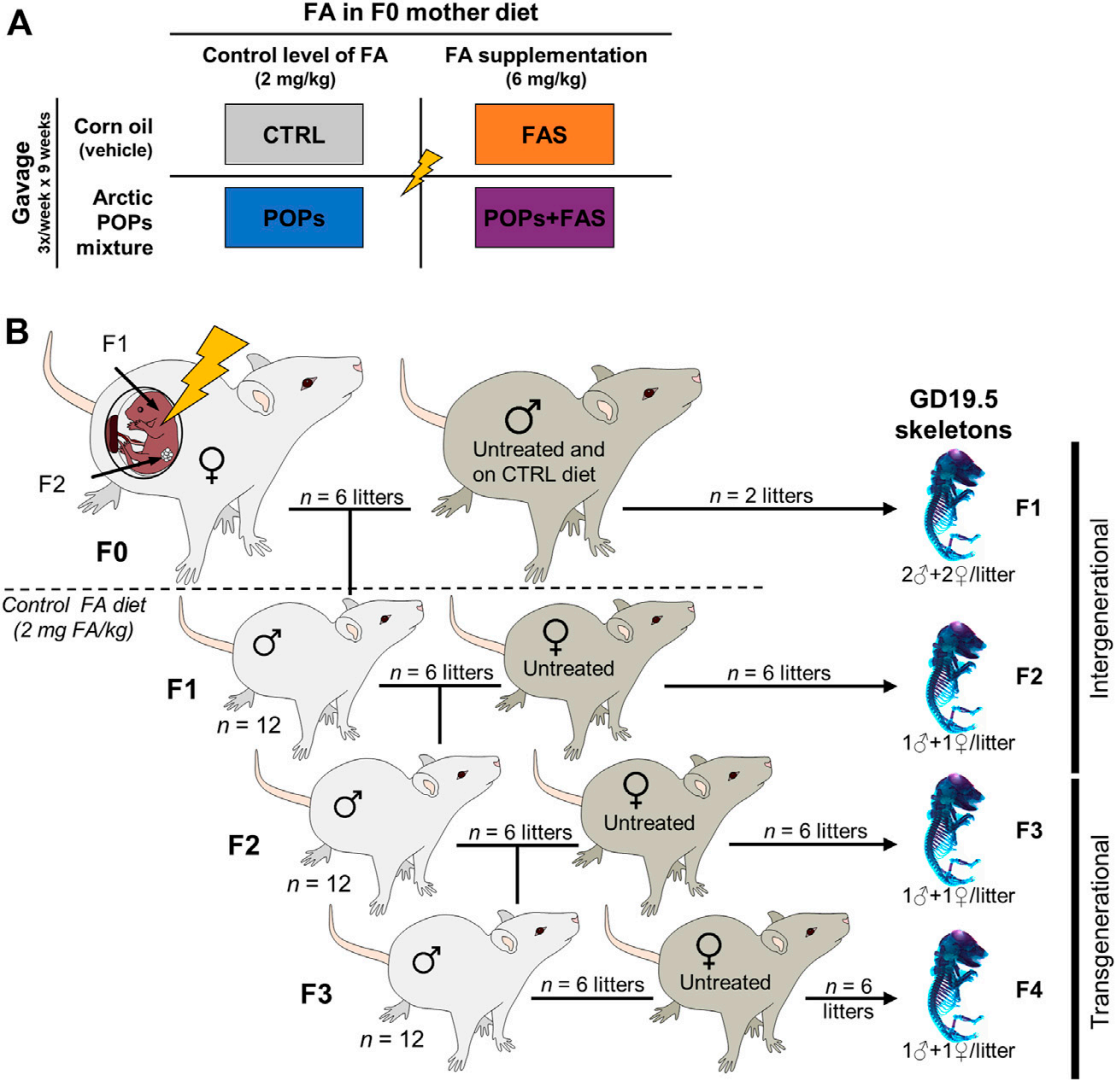
Experimental design. **(A)** Description of the four treatments groups of Sprague-Dawley F0 founder females, following a 2 × 2 factorial design: two factors and two doses. The first factor is the environmentally relevant Arctic POPs mixture, administrated by gavage trice weekly, and is either present in corn oil (POPs) or absent (vehicle only). The second factor is the folic acid (FA) content of the AIN-93 diets given *ad libitum*. The F0 females received either a FA control diet (2 mg FA/kg diet), or a FA-supplemented diet (6 mg FA/kg diet). CTRL = control group (FA control diet, without POPs); POPs = POPs exposed group (FA control diet, with POPs); FAS = FA supplemented group (FA-supplemented diet, without POPs); POPs + FAS = POPs-exposed and FA supplemented group (FA-supplemented diet, with POPs). **(B)** Schematic representing the four generation Sprague-Dawley rat model. F0 founder females (*n* = 8), were randomly assigned to one of the treatment groups and treated accordingly for 5 weeks before mating to an untreated male (FA control diet, without POPs) and until parturition. Twelve of the resulting F1 males were mated to untreated females to produce F2 rats and so on until F4. At each generation, fetuses at GD19.5 (males and females) were collected to assess skeleton development. After the birth of the F1 litters, the F0 mothers, and subsequent generation received the FA control diet and no POPs. F0 to F4, generations F0 to F4; POPs, persistent organic pollutants; FA, folic acid; GD19.5, Gestational Day 19.5.

All animal protocols were conducted in accordance with the guidelines of the Canadian Council on Animal Care and approved by the Université Laval Animal Research Ethics Committee (certificate # 2015010-2) as described previously (Herst et al., 2019; Lessard et al., 2019; Navarro et al., 2019). Thirty-two Sprague-Dawley female rats (aged 45 days and weighing 140–180 g) (Charles River Laboratories; Saint-Constant, Quebec, Canada) constituted the F0 founder dams and were pair-housed in standard cages at 22°C (50% humidity) with 12-h light-dark cycle. Food and water were provided *ad libitum*. After 10 days of acclimatization, the F0 females were randomly assigned to one of four treatments groups (Figure 1A): 1) the control group (“CTRL”; *n* = 8) was gavaged with corn oil (no POPs) and fed the control FA diet; 2) the POPs-treated group (“POPs”; *n* = 8) was gavaged with the POPs mixture and fed the control FA diet; 3) the no POPs-FA supplemented group (“FAS”; *n* = 8) was gavaged with corn oil and fed the FA supplemented diet; 4) the POPs-treated and FA-supplemented group (“POPs + FAS”; *n* = 8) was gavaged with the POPs mixture and fed the FA supplemented diet.

The F0 females were weighed and gavaged thrice weekly with corn oil (CTRL and FAS groups) or the POPs mixture (POPs and POPs + FAS groups) for 5 weeks before mating with untreated males (no POPs, control FA diet), aged 90 days and weighing 200–240 g (Charles River Laboratories). Mating was confirmed by the presence of spermatozoa in vaginal smears, which were performed every morning, and the day a female was found sperm-positive was designated Gestational Day 0.5 (GD 0.5). Pregnant females were housed individually, and gavaged trice weekly until parturition. After the birth of the F1 litters, all F0 females and subsequent generations received the FA control diet. At Postnatal Day (PND) 21, the pups were weaned, and 12 randomly selected males (two for each of the six F0 dam that gave birth) were kept and pair-housed (a schematic of this experimental design is shown in Figure 1B).

At PND 90, F1 males were mated with untreated (no POPs, FA control diet) nulliparous and sexually mature females (aged 63–74 days, Charles River Laboratories) to generate F2 (Figure 1B). The F3 and F4 lineages were generated in the same manner. At GD 19.5, females were sacrificed to collect fetuses (F1: *n* = 2 pregnant females per group; F2-F3-F4: *n* = 6 pregnant females per group) and the remaining females gave birth (*n* = 6).

In the current study, F0 mothers received the interventions during pregnancy, therefore the F1 and F2 pups were directly exposed (intergenerational exposure), through the placenta and lactation for the F1 pups, and through the germ cells of the F1 fetuses for the F2 pups. The F3 and F4 generations, however, were not exposed to the treatment and will hereafter be referred to as transgenerational exposure.

### Euthanasia and fetal skeleton preparation

Dams were anesthetized with 3% isoflurane, then euthanized by exsanguination via cardiac puncture and CO_2_ asphyxiation. The fetuses were collected by caesarean-section at GD19.5. This time point was chosen to ensure that malformed fetuses were assessed that could have otherwise been cannibalized at birth by the dam. Eight fetuses per group (2♂ and 2♀ each from 2 different litters) were collected in F1, and 12 fetuses per group (1♂ and 1♀ each from 6 different litters) were collected in F2-F3-F4. Fetuses were sexed by measuring the anogenital distance with an electronic micrometer, then confirmed by the presence of testes or ovaries after abdominal dissection. The fetuses were placed in 95% ethanol at room temperature for maceration and storage until staining.

### Double skeletal staining

Double staining of the fetal bone and cartilage was performed with alcian blue and alizarin red S stains. Our protocol was adapted for rats by empirically modifying dye concentration, incubation time and KOH concentration from previously described procedures (Menegola et al., 2001; Lambrot et al., 2013; Rigueur and Lyons, 2014). Briefly, fetuses (GD19.5) were removed from 95% ethanol and put in a warm bath for up to a minute to facilitate removal of the skin, muscles, and viscera. The cleared skeletons were placed in 100% acetone overnight, and then stained with alcian blue solution 0.03% (80% ethanol, 20% acetic acid) for 12 h. The fetuses were washed in 70% ethanol to remove the blue dye and stored in 95% ethanol overnight. The skeletons were then transferred into a 2% KOH solution for 24 h, after which they were stained in the alizarin red S solution 0.005% (KOH 1%) for 4 h. The double-stained skeletons were stored in a glycerin and 70% ethanol solution (1:1).

### Quantitative assessment of skeletal ossification and measurement of skeletal structures

The degree of ossification and, where possible, bone lengths were quantitatively assessed for skeletal sections (head, sternum, vertebrae, and limbs). Data were scored based on Kaufman (1994) and Menegola et al. (2001), and presented as a percentage (total score per section/maximum score). Analysis of the head ossification was divided into three measures: the skull bones (frontal, parietal, interparietal, supraoccipital, exoccipital, and basioccipital), the mandible and the premaxilla. Sternum development was evaluated by the degree of sternebrae ossification. The spinal evaluation comprised the cervical, thoracic, lumbar, and sacral vertebrae. Both front and hind paws were examined at distal, intermediate, and proximal phalanges. The metacarpals for the front paws and the metatarsals for the hind paws were evaluated. The front and hind paws, each composed of five digits, were numbered from 1 to 5, the thumb being 1 and the smallest digit being 5. The analysis was based on the average of left and right front and hind paws for each individual.

**FIGURE 2:**
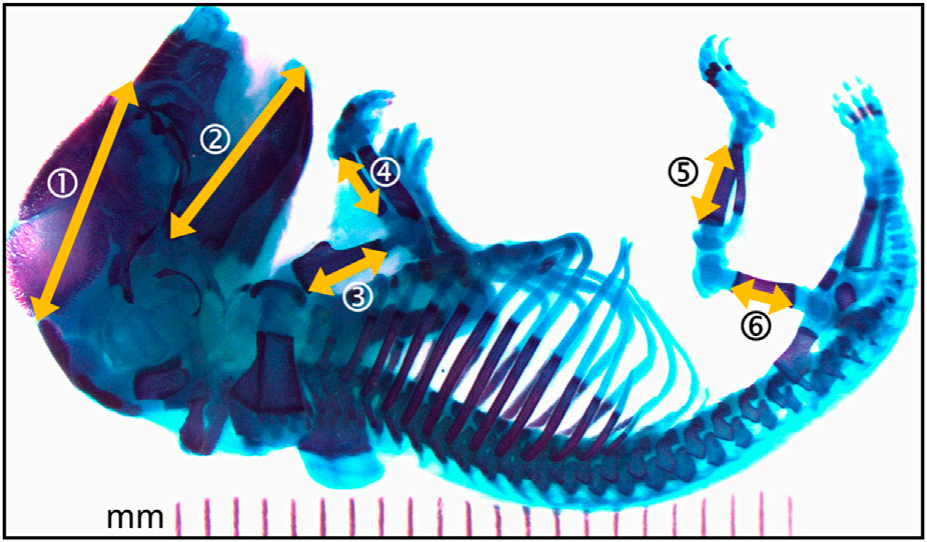
Photomicrograph of the full skeleton following double staining with alcian blue and alizarin red S. Bone is stained purple and cartilage, blue. Yellow arrows represent the structural distances that where measured. 1- craniofacial length; 2- mandible length; 3- humerus length; 4- radius length; 5- femur length; 6- tibia length. Left and right sides where measured when applicable.

The bone lengths were measured with ImageJ (Image Processing and Analysis in Java, public domain, version 1.52 m) as shown in Figure 2. These included measures of head structures (craniofacial distance and mandible length) and the length of the main long bones (humerus, radius, femur, and tibia).

### Statistical analysis

For each treatment group, data are expressed as mean ± standard error of the mean (SEM). One-way analysis of variance (ANOVA) for a 2 × 2 factorial design using the MIXED procedure in SAS^®^ University 9.4 Edition (SAS Institute Inc., Cary, United States) was performed. The dam effect (F0 founder females and subsequent F1-F2-F3 dams), the sex of the pups and the number of pups per litter were controlled in the model. When the data showed non-significant sex effects, male and female data were combined. Throughout the four generations, male lineages were derived from an equivalent number of the F0 founder females to increase diversity and minimize the effect of the F0 dams. The main effects of POPs (in POPs and POPs + FAS groups) and FAS (in FAS and POPs + FAS groups) as well as their interaction (POPs*FAS) were determined. If there was no significant interaction, the main effect of the POPs and/or FAS were interpreted individually. The assessment of the skeletal ossification and structural measurements were conducted using a blind analysis.

## Results

### Both prenatal and ancestral persistent organic pollutants exposure reduce ossification

**FIGURE 3:**
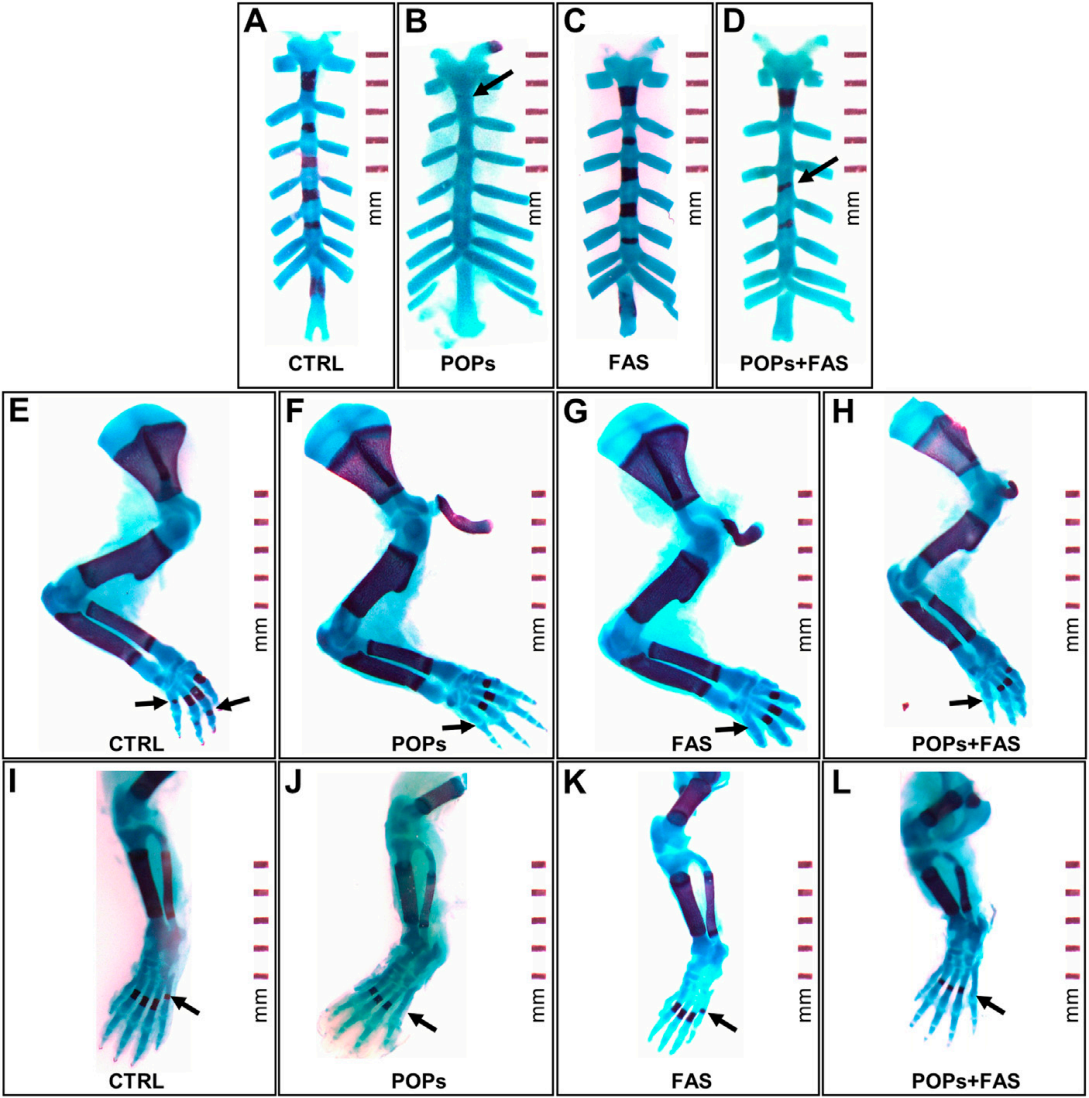
Examples of impaired skeletal ossification in rat fetuses exposed to POPs and FA. Skeletal double staining procedure with alcian blue and alizarin red S was performed in all treatment groups. Bone is stained purple and cartilage, blue. **(A–D)** Examples of POPs-induced incomplete **(B)** or misaligned **(D)** development ossification of the sternebrae plates in the POPs and POPs + FAS groups as shown by the arrows. **(E–H)** Examples of POPs and FAS-induced retarded ossification of the forelimb digits (arrows) in the POPs, FAS and POPs + FAS groups compared to the CTRL group **(E)**. **(I-L)** Examples of POPs-induced delayed ossification of the hindlimb digits (arrows) in the POPs and POPs + FAS groups compared to the CTRL group **(I)**. CTRL, control group (base FA diet, without POPs); POPs, POPs exposed group (base FA diet, with POPs); FAS, FA supplemented group (FA-supplemented diet, without POPs); POPs + FAS = POPs-treated and FA supplemented group (FA-supplemented diet, with POPs). F0 to F4, generations F0 to F4; POPs, persistent organic pollutants; FA, folic acid; GD19.5, Gestational Day 19.5.

To determine if maternal FAS can protect against the expected negative impacts of prenatal and ancestral paternal POPs exposure on the bone ossification of four generations of rat fetuses, a quantitative assessment of the degree of skeletal ossification was performed. The number of fetuses obtain throughout the four generations were always between 8 and 18 per litter, with a mean of 13,2 fetuses per litter (Table 2). The results are presented in Table 3; Figure 3 shows examples of reduced ossification in the sternum, front paws, and hind paws in the POPs-exposed and/or FAS-treated groups. No difference was noted regarding the ossification profile between males and females in any generation. There was no difference in the degree of ossification of the vertebral column among the four treatment groups within any generation. The ossification of the sternum (*p* = 0.05) and the front (*p* = 0.05) and (*p* = 0.03) hind paws, specifically at the digits, is reduced in POPs-exposed F1 fetuses, with no beneficial effect of FAS. Indicating a specific effect of folic acid supplementation alone, the front paws only of FAS-treated F1 fetuses present a similar decreased ossification (*p* = 0.003) as the POPs-treated groups. Reduced ossification induced by POPs exposure in the sternum (*p* = 0.04) persisted in the F2 generation. No significant differences in ossification were noted in the F3-F4 fetuses.

**TABLE 2:** Number of pups per litter across the four generations.

	CTRL			POPs			FAS			POPs + FAS		
	Mean	Min	Max	Mean	Min	Max	Mean	Min	Max	Mean	Min	Max
F1 (*n* = 2 l)	14.5	14.0	15.0	14.0	13.0	15.0	14.0	12.0	16.0	14.0	14.0	14.0
F2 (*n* = 6 l)	12.5	10.0	15.0	13.2	9.0	15.0	14.0	13.0	18.0	13.0	12.0	14.0
F3 (*n* = 6 l)	11.6	10.0	14.0	13.8	13.0	14.0	12.8	10.0	15.0	12.0	10.0	13.0
F4 (*n* = 6 l)	13.8	10.0	18.0	12.3	8.0	15.0	13.0	11.0	15.0	12.0	10.0	14.0

**TABLE 3:** Bone ossification of rat fetuses across four generations following prenatal or ancestral exposure to POPs and/or FAS through the paternal lineage.

	F1^a^				*p*-values		
	CTRL	POPs	FAS	POPs + FAS	POPs	FAS	POPs*FAS
Head (%)	92.9 ± 1.8	91.1 ± 1.8	91.8 ± 1.8	92.2 ± 1.8	0.68	0.98	0.54
Sternum (%)	72.8 ± 7.0	56.1 ± 6.9	73.9 ± 6.9	63.2 ± 6.9	**0.05**	0.55	0.67
Front paws (%)	46.6 ± 2.5	36.9 ± 2.5	34.0 ± 2.5	33.4 ± 2.5	**0.05**	**0.003**	0.08
Hind paws (%)	32.2 ± 0.7	30.2 ± 0.7	32.3 ± 0.7	31.3 ± 0.7	**0.03**	0.37	0.50

	F1^b^				*p-*values		
	CTRL	POPs	FAS	POPs + FAS	POPs	FAS	POPs*FAS
Head (%)	93.4 ± 1.3	92.1 ± 1.2	91.8 ± 1.2	92.1 ± 1.3	0.71	0.52	0.51
Sternum (%)	64.7 ± 6.6	47.5 ± 6.2	54.3 ± 6.4	45.2 ± 6.8	**0.05**	0.33	0.54
Front paws (%)	35.7 ± 0.9	34.0 ± 0.9	35.5 ± 0.9	33.8 ± 0.9	0.06	0.83	0.97
Hind paws (%)	35.7 ± 0.7	35.1 ± 0.6	34.8 ± 0.6	33.7 ± 0.7	0.18	0.09	0.73

	F1^c^				*p-*values		
	CTRL	POPs	FAS	POPs + FAS	POPs	FAS	POPs*FAS
Head (%)	94.5 ± 1.3	94.2 ± 1.5	94.5 ± 1.3	92.9 ± 1.2	0.48	0.60	0.66
Sternum (%)	66.0 ± 7.4	62.7 ± 7.5	63.9 ± 7.5	63.9 ± 6.7	0.82	0.94	0.84
Front paws (%)	33.4 ± 0.4	33.7 ± 0.4	33.3 ± 0.4	33.8 ± 0.4	0.31	0.97	0.73
Hind paws (%)	30.7 ± 0.8	31.0 ± 0.8	31.0 ± 0.8	31.2 ± 0.7	0.73	0.71	0.91

	F4^d^				*p-*values		
	CTRL	POPs	FAS	POPs + FAS	POPs	FAS	POPs*FAS
Head (%)	94.9 ± 1.8	96.2 ± 1.5	94.5 ± 1.6	94.8 ± 1.7	0.17	0.69	0.25
Sternum (%)	54.8 ± 3.8	54.5 ± 3.5	52.1 ± 3.5	62.2 ± 4.1	0.06	0.07	0.95
Front paws (%)	34.3 ± 05	33.3 ± 0.5	33.7 ± 0.5	33.4 ± 0.5	0.20	0.60	0.48
Hind paws (%)	29.2 ± 0.6	30.7 ± 0.5	30.6 ± 0.5	31.1 ± 0.6	0.08	0.09	0.38

The *p*-value were calculated by ANOVA for a 2 × 2 factorial design using the MIXED procedure. Results are expressed as mean ± SEM. Values in bold are statistically significant.

a *n* = 8 fetuses (2♂+2♀ from each of 2 l).

b *n* = 12 fetuses (1♂+1♀ from each of 6 l).

c *n* = 12 fetuses (1♂+1♀ from each of 6 l).

d *n* = 12 fetuses (1♂+1♀ from each of 6 l).

### Persistent organic pollutants exposure modifies bone lengths intergenerationally

**FIGURE 4:**
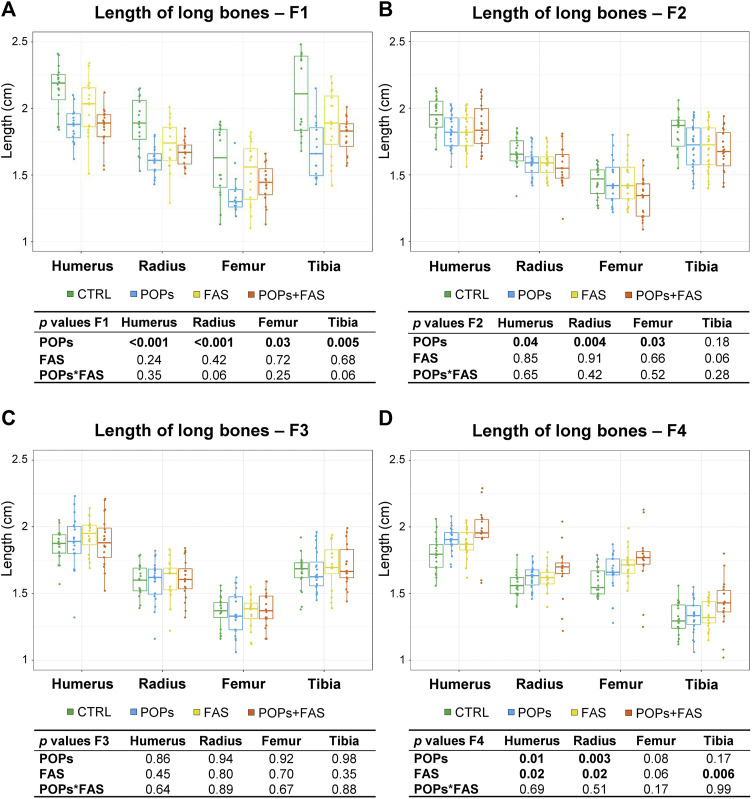
Prenatal and ancestral paternal POPs exposure results in shorter long bones in GD19.5 fetuses of F1 generation **(A)** and of F2 descendants **(B)**. No difference in the lengths of the long bones in the F3 generation **(C)**, but longer long bones observed in the F4 descendants were observed **(D)**. The lengths, in mm, of bilateral long bones of the forelimbs (humerus, radius) and hindlimbs (femur, tibia) were measured and are expressed as means ± SEM. Eight fetuses per group (2♂ and 2♀ each from 2 different litters) were collected in F1, and 12 fetuses per group (1♂ and 1♀ each from 6 different litters) were collected in F2-F3-F4. The *p*-value tables under each histogram present the main effect of the POPs exposure (POPs in POPs and POPs + FAS groups), the main effect of the FAS treatment (FAS in FAS and POPs + FAS groups) and the interaction between the POPs and FAS treatments (POPs*FAS). CTRL, control group (base FA diet, without POPs); POPs, POPs exposed group (base FA diet, with POPs); FAS, FA supplemented group (FA-supplemented diet, without POPs); POPs + FAS, POPs-exposed and FA supplemented group (FA-supplemented diet, with POPs). F0 to F4, generations F0 to F4; POPs, persistent organic pollutants; FA, folic acid; GD19.5, Gestational Day 19.5.

Head structure and long bone lengths were measured to evaluate the consequence of exposure to POPs and/or FAS treatment on bone growth (Table 4; Figure 4). No difference was noted in bone structure measures between males and females in any generation.

**TABLE 4:** Head structure lengths of rat fetuses across four generations following prenatal or ancestral exposure to POPs and/or FAS through the paternal lineage.

Length (mm)	F1^a^				*p*-values		
	CTRL	POPs	FAS	POPs + FAS	POPs	FAS	POPs*FAS
Mandible	5.86 ± 0.15	5.39 ± 0.15	5.27 ± 0.15	5.72 ± 0.15	0.94	0.40	**0.004**
Craniofacial	9.50 ± 0.17	9.37 ± 0.17	9.28 ± 0.17	9.31 ± 0.17	0.77	0.41	0.65

	F2^b^				*p*-values		
	CTRL	POPs	FAS	POPs + FAS	POPs	FAS	POPs*FAS
Mandible	5.47 ± 0.09	5.37 ± 0.08	5.55 ± 0.09	5.27 ± 0.09	**0.03**	0.89	0.32
Craniofacial	9.03 ± 0.10	8.81 ± 0.09	8.92 ± 0.10	8.76 ± 0.10	0.06	0.46	0.77

	F3^c^				*p*-values		
	CTRL	POPs	FAS	POPs + FAS	POPs	FAS	POPs*FAS
Mandible	5.60 ± 0.13	5.62 ± 0.14	5.57 ± 0.13	5.62 ± 0.12	0.80	0.86	0.91
Craniofacial	8.77 ± 0.16	8.73 ± 0.17	9.01 ± 0.16	9.06 ± 0.14	0.95	0.16	0.78

	F4^d^				*p*-values		
	CTRL	POPs	FAS	POPs + FAS	POPs	FAS	POPs*FAS
Mandible	5.49 ± 0.12	5.59 ± 0.11	5.46 ± 0.11	5.62 ± 0.12	0.26	0.97	0.81
Craniofacial	8.90 ± 0.10	9.20 ± 0.09	9.13 ± 0.09	9.06 ± 0.10	0.24	0.64	0.71

The *p*-value were calculated by ANOVA for a 2 × 2 factorial design using the MIXED procedure. Results are expressed as mean ± SEM. Values in bold are statistically significant.

a *n* = 8 fetuses (2♂+2♀ from each of 2 l).

b *n* = 12 fetuses (1♂+1♀ from each of 6 l).

c *n* = 12 fetuses (1♂+1♀ from each of 6 l).

d *n* = 12 fetuses (1♂+1♀ from each of 6 l).

In F1 fetuses, there was an interaction between POPs and FAS (POPs*FAS *p* = 0.004), where the POPs exposure reduced the length of the mandible, but the co-intervention with FAS resulted in lengths similar to the CTRL measurements. As for long bone length, a similar pattern of reduction was observed in the humerus (*p =* 0.001), radius (*p* = 0.003), femur (*p =* 0.03), and tibia (*p* = 0.005) following POPs exposure.

In F2 fetuses, the mandible (*p* = 0.03) length was smaller in the POPs-treated lineages. Similar to the F1 generation, POPs exposure also reduced the length of the F2 fetal humerus (*p =* 0.05), radius (*p* = 0.005), and femur (*p =* 0.03), without effect on the tibia. Furthermore, the co-intervention of FAS did not counteract the consequences of POPs exposure. Similar to the bone ossification results, there was no difference in the lengths of head structures or in the lengths of the long bones in the F3 generation.

In the F4 generation, the POPs ancestral exposure resulted in longer humerus (*p =* 0.01) and radius (*p* = 0.003), contrasting with the reductions seen in F1 and F2. Moreover, ancestral FAS treatments had the same increasing effect as the POPs for the humerus (*p =* 0.02) and radius (*p* = 0.02).

## Discussion

We studied an environmentally relevant mixture to assess the developmental consequences of multiple POPs exposure at environmentally-relevant levels. Exposure to low doses of toxicants during critical developmental windows, such as *in utero* and during lactation, has been shown to have a negative impact on the health of individuals (Kortenkamp, 2008; Mocarelli et al., 2011; Li et al., 2020; Malaisé et al., 2020). Due to its capacity to produce hormones, bone is an active and dynamic regulator of whole-body homeostasis, and alteration of normal bone development could therefore also affect other systems (Karsenty and Ferron, 2012). Herein we demonstrated that prenatal paternal exposure to an environmentally-relevant mixture of POPs impairs fetal skeletal development. We also assessed both male and female fetuses following paternal prenatal exposure to POPs, however, no significant difference was observed due to fetal sex in any generation, although sex-specific responses to the environment are well-established, especially after dioxin and/or dioxin-like exposure (Wejheden et al., 2010; Institute of Medicine, 2011; Golden and Voskuhl, 2017). Moreover, abnormal ossification was evident in subsequent, unexposed generations up to the F2. Our hypothesis that FAS could palliate any adverse effects of POPs exposure was justified by our previous report showing that POPs-induced sperm epigenetic alterations were partly corrected by FAS (Herst et al., 2019).

### Direct exposure to persistent organic pollutants affects fetal skeleton over multiple generations

Our data show that direct exposure to POPs resulted in delayed ossification of the sternum (F1-F2) and front and hind paws (F1). At GD19.5, the sternum is expected to be only partially ossified, but ossification centers are usually present in most sternebrae (Menegola et al., 2001). Exposure to our POPs mixture resulted in a poorly ossified sternum over two generations (F1-F2). Another study reported a similar phenotype in the first generation of late term rat fetuses perinatally exposed to an environmentally relevant mixture of brominated flame retardants (BFRs), including polybrominated diphenyl ethers (PBDEs), that are also POPs (Berger et al., 2014).

Direct exposure to POPs also affected the length of the limb long bones over multiple generations (F1-F2; F4) as it resulted in shorter humerus, radius and femur bones in F1 and F2 fetuses and in F1 tibia. Surprisingly, F4 fetuses with ancestral POPs exposure had longer front limb long bones. Previous studies demonstrated bone alterations related to POPs exposure up until only the first generation. Indeed, perinatal exposure to a similar environmentally-relevant POPs mixture including methylmercury, resulted in delayed F1 femur growth and ossification, and reduced mechanical strength in a rat model (Elabbas et al., 2011a). The same team further explored the specific role of PCBs in their mixture, namely Aroclor 1254, a technical mixture of several PCBs. They observed the same consequences as with the full mixture, suggesting that bone-related consequences could be attributed to the PCBs in the POPs mixture (Elabbas et al., 2011b). Indeed, certain PCBs are *dioxin-like* and can mimic the action of dioxins (Giesy and Kannan, 1998), which have been previously shown to be detrimental to bone health (Jämsä et al., 2001). Although the mechanisms of bone toxicity caused by dioxin-like chemicals are not totally understood, studies suggest that they impair the normal function of the aryl hydrocarbon receptor (AhR), implicated in bone remodelling and general bone homeostasis (Herlin et al., 2010; Yu et al., 2018). The anti-estrogenic PCB 126 has also been linked to impaired mineralisation and reduced length of long bones (Lind et al., 1999; Lind et al., 2000).

Furthermore, dissecting the impacts of each component suggest that the mixture used here can perturb several hormone-signalling pathways because of its: 1) estrogenic activity, due to aldrin, dieldrin, DDT/DDE, B-HCH, and toxaphene; 2) strong antiandrogenic activity, due to PCBs and DDT/DDE compounds blocking the androgen receptor; 3) dioxin-like activity, due to certain PCBs (Donaldson et al., 2010). Androgen hormones have been shown to modulate longitudinal growth in long bones (Wiren, 2005). Androgen receptors have been found on osteoblasts, osteocytes, and chondrocytes which further implies a direct role of androgen on bone and cartilage (Chen and Guo, 2013). The strong inhibition of the androgen receptor by the Arctic POPs mixture could explain why the growth of the long bones was impaired in this model. Future investigation should assess the interaction among these toxicants, the endocrine system, and bone development.

Although these endocrine disrupting mechanisms could partly explain the bone variations observed in F1, it is not clear how the paternal exposure to POPs and FAS influences the subsequent, non-directly exposed generations. An increasing body of literature demonstrates that the paternal environment can affect the development of his offspring over several generations through epigenetic perturbations of the male germline (Lambrot et al., 2013; Braun et al., 2017; Ly et al., 2017). Our team previously demonstrated that prenatal and ancestral POPs exposure led to intergenerational alterations of specific sperm miRNA profiles, known to target genes implicated in embryonic organ development, among others (Herst et al., 2019). We also observed reduced sperm parameters throughout three unexposed generations following prenatal and ancestral POPs treatments (Lessard et al., 2019). The increased long bone lengths in POPs-exposed F4 fetuses reported in the present study is consistent with our previous surprising observation that the most differentially expressed genes in two-cell embryos and the poorest pregnancy outcomes also occurred in the F4 litters (Lessard et al., 2019). Indeed, environmentally sensitive epigenomic regions have been identified in human sperm that could be transmitted to subsequent generations (Chan et al., 2019). Using an inheritance simulation statistical package to assess the rat sperm DNA methylome demonstrated that early-life paternal exposure to the same POPs mixture used in this study leads to inherited methylation changes that are inter- and transgenerationally transmitted (Belleau et al., 2018).

In this study, following our various publications, we hypothesized that the phenotypes observed at the bone level could potentially be the result of alterations in the sperm epigenome, including the methylome and miRNAs. In this same vein, another potential mechanism that was not explored in this study is the role of the stress hormone corticosterone. This hormone known as a modulator of transgenerational epigenetic inheritance could be an adjacent or complementary mechanism to POPS-induced changes observed across generations (Short et al., 2016; Yeshurun et al., 2017).

### Folic acid supplementation did not prevent bone impairment associated with persistent organic pollutants exposure

Maternal supplementation of FA is recommended to ensure adequate fetal development (Antony, 2007). The placenta concentrates folate into the fetal circulation and the maternal-to-fetal folate transfer is mediated by placental folate receptors (Henderson et al., 1995; Antony, 2007). Sufficient folate *in utero* is essential for the neuroskeletal development of the fetus, where the brain, skull and spine are growing interdependently (Morse, 2012). Osteogenesis takes place during an intensive cell proliferation and DNA synthesis period, which involves one-carbon metabolism where folate acts to carry and donate one-carbon units to support biosynthetic reactions including for *de novo* nucleotide and methionine synthesis (Crider et al., 2012). Therefore, as skeletal development occurs mainly during gestation and early life, we hypothesized that prenatal folic acid supplementation (FAS) would reduce and/or counteract the harmful bone effects induced by POPs assuming that they were related to cell proliferation and differentiation.

Our team previously reported some protective effect of FAS on sperm parameters and sperm miRNA profiles across multiple generations (Herst et al., 2019; Lessard et al., 2019). In this study, however, we observed few noticeable protective effects of direct or ancestral FAS exposure on skeletal development. Prenatal dietary FAS did not counteract delayed ossification and reduced length of long bones in fetuses exposed to POPs in any generation. Moreover, FAS alone led to a similar phenotype to POPs exposure in the F1 generation, with decreased ossification of the front paws. FAS also led to negative effects on pregnancy outcomes, namely fertility rates and preimplantation losses in F3 fathers (Lessard et al., 2019). Our team also showed that FAS could not prevent the deleterious consequences of POPs exposure on lipid homeostasis in male descendants (Navarro et al., 2019). Taken together, therefore, FAS inconsistently prevents the negative effects associated with POPs exposure that occurred over several generations. The timing of exposure may have been insufficient since the FAS lasted only as long as direct exposure to POPs. It is also possible that FA sufficiency, as represented by the control diet, provided a maximal protective effect against POPs and therefore FAS provided no additional benefits. It is possible that a more severe phenotype would have been observed in rats exposed to POPs and a FA-insufficient diet.

## Conclusion

In conclusion, this study identifies the skeleton as a susceptible organ system to POPs exposure during the perinatal period, with multigenerational skeletal phenotype alterations. The phenotypes observed were modest, but they reflect the consequences of real-life exposure levels. Indeed, the environmentally relevant POPs mixture is designed to approximate the body burdens observed in human populations in northern Canada (Anas et al., 2005) and the dietary FA levels are physiological, and approximate a level of FA intake achievable by women consuming prenatal supplements and FA fortified foods (Swayne et al., 2012b). The nutritional solution tested here did not successfully counteract the effects of POPs exposure, but a lifetime exposure or other nutritional strategies may warrant investigation. Nevertheless, the consequences of POPs exposure are not only persisting throughout the individuals’ lifetime, but also over generations. This is of importance, as human populations and wildlife living in regions that are persistently contaminated with these environmental toxicants show impaired reproductive functions and increased adverse pregnancy outcomes (Sonne, 2010; Auger et al., 2013; Vested et al., 2014). This research thus stresses the importance of preventing exposure to POPs and finding an accessible, protective alternative with positive health implications for Inuit and their children.

## Data Availability

The raw data supporting the conclusion of this article will be made available by the authors, without undue reservation.
